# Usefulness of BATF3 Immunohistochemistry in Diagnosing Classical Hodgkin Lymphoma

**DOI:** 10.3390/diagnostics11061123

**Published:** 2021-06-20

**Authors:** Julian Benckendorff, Johanna Kuchar, Frank Leithäuser, Malena Zahn, Peter Möller

**Affiliations:** 1 Department of Pathology, University Hospital Ulm, Albert-Einstein-Allee 23, 89081 Ulm, Germany; frank.leithaeuser@uniklinik-ulm.de (F.L.); malena.zahn@uniklinik-ulm.de (M.Z.); peter.moeller@uniklinik-ulm.de (P.M.); 2 Department of Vascular and Endovascular Surgery, University Hospital Augsburg, Stenglinstraße 2, 86156 Augsburg, Germany; Johanna.Kuchar@uk-augsburg.de

**Keywords:** classical Hodgkin lymphoma 1, BATF3 2, immunohistochemistry 3

## Abstract

It is well recognized that the AP-1 transcription factor BATF3 is constitutively expressed in Hodgkin/Reed-Sternberg (HRS) cells, but its potential as a diagnostic marker for classical Hodgkin lymphoma (cHL) has not yet been addressed. In this study, we performed immunohistochemistry and analyzed the BATF3 expression in lymphoma cells on 218 lymphoma samples belonging to 14 different lymphoma entities. We observed varying degrees of BATF3 expression in nearly half of the cases (*n* = 100) with BATF3 expression being a constitutive feature of cHL (*n* = 53) and anaplastic large cell lymphoma (ALCL). By scoring BATF3 expression (BATF3-score) we observed constitutively high BATF3-scores in cHL and ALCL and low to moderate BATF3-scores in all other entities examined. Western blot analysis confirmed BATF3 protein expression in cell lysates from cHL cell lines (*n* = 7). Thus, BATF3 can be considered a useful IHC marker for the diagnosis of cHL as it is highly sensitive and sufficiently specific when analyzed by BATF3-scoring.

## 1. Introduction

Classical Hodgkin lymphoma (cHL) is one of the most common lymphomas [1]. The correct diagnosis requires histopathological examination of tumor infiltrated tissue and detection of the characteristic tumor cells, the Hodgkin/Reed-Sternberg (HRS) cells. HRS cells are large mono- and multinuclear cells with a characteristic morphology and immunophenotype that result from the neoplastic transformation of a preapoptotic germinal center B-cell [2,3]. Diagnosis can be complicated by the fact that HRS cells are typically rare and distributed over a large inflammatory background. The differential diagnosis is broad and includes non-neoplastic changes, Non-Hodgkin lymphomas (NHLs) and nodular lymphocyte predominant Hodgkin lymphoma (NLPHL). Therefore, immunophenotyping of HRS cells is widely used to facilitate cHL diagnosis. 

There is no specific immunohistochemical (IHC) marker for cHL. The immunophenotype of HRS cells is characterized by a substantial loss of B-cell associated antigens [4,5,6,7,8] and by an aberrant expression of markers that are usually associated with other hematopoietic lineages [9,10,11]. In typical cases, identification of HRS cells is based on detection of CD30 [12], CD15 [13], IRF4 [14] and Pax5 [15] in combination with a loss of CD20 and CD79a [5]. Diagnostic difficulties may arise in non-lymphoid tissue or in cases where HRS cells present with an atypical immunophenotype [16]. In these cases, ancillary IHC markers can help to establish the cHL diagnosis.

Published studies indicate that BATF3 expression is a distinctive feature of HRS cells [17,18,19,20,21,22,23,24]. BATF3 is a member of the AP-1 family of transcription factors that is normally expressed in a subset of T helper type 1 cells and conventional dendritic cells (cDCs) [25,26]. There is no BATF3 expression in normal B-cells with the exception of CD30-positive B-cells of reactive lymph nodes [20]. In cHL, BATF3 expression is promoted by the constitutive activity of the JAK/STAT signaling pathway [20]. The resulting BATF3/JUN dimers bind to MYC, induce MYC expression and thereby contribute to proliferation and survival of the HRS cells [20]. 

Since BATF3 is constitutively expressed in HRS cells, we hypothesized that BATF3 could be an ancillary IHC marker for the diagnosis of cHL. To test this, we performed BATF3 immunohistochemistry and compared the BATF3 expression in cHL to that in NLPHL and NHL.

## 2. Materials and Methods

### 2.1. Tissue and Cell Lines

Specimens were drawn from our archive of formalin fixed paraffin embedded (FFPE) tissue. Hematoxylin-eosin stained sections and available immunohistochemical and molecular material were checked and, if necessary, completed by newly performed staining. A total of 218 samples were selected. FFPE material was pseudonymized to comply with the German law for correct usage of archival tissue for clinical research [27]. The samples included 53 cHL, 16 NLPHL and 149 NHL. Antibody validation was performed by Western blotting. Cell lines of cHL (L-428, L-1236, U-HO1, HDLM-2, KM-H2, Sup-HD1, L-540), Burkitt lymphoma (BL) (Ramos) and T-cell lymphoblastic lymphoma/Leukemia (T-LBL/ALL) (Jurkat) were selected. All cell lines were obtained from Leibniz Institut DSMZ (Braunschweig, Germany) apart from U-HO1 that we generated [28]. The study was approved by the Ethics Committee of Ulm University (Biomaterialstudien Langzeit-Archiv der Pathologie, protocol code: 276/13, date: 9 September 2013).

### 2.2. Immunohistochemistry

BATF3 staining was performed on 2-3 µm thick sections of FFPE tissue on DAKO Autostainer PLUS (Agilent, Santa Clara, CA, USA). Antigen retrieval was performed by microwave heating in citrate buffer, pH6, for 20 min. After peroxidase blocking, sections were incubated for 1 hour with a polyclonal sheep anti-BATF3 antibody (AB) (R&D Systems, Minneapolis, MN, USA) at a dilution of 1:200. Polink-2 HRP Plus Sheep IgG DAB Detection System (GBI Labs, Bothell, WA, USA) was used for signal amplification. 

BATF3 expression was scored in analogy to the H-score method (BATF3-score) by multiplying the percentage of stained tumor cells by the staining intensity [29]. The range of possible scores was from 0 to 300. In cases where tissue quality interfered with IHC staining quality, we compared the staining intensity of the tumor cells to the staining intensity of the BATF3-expressing immune cells within the tumor microenvironment [25,26]. The latter served as internal controls to further optimize the calibration of the score. Expression level of each lymphoma entity was rated as low, moderate or high according to the mean value of the BATF3-score. 

### 2.3. Western Blotting

Proteins were separated by 10% SDS-PAGE and transferred to a 0.2 mm nitrocellulose membrane (GE Healthcare, Chicago, IL, USA). Western blotting was performed using sheep anti-BATF3 AB (R&D Systems) and mouse anti-β-actin AB (Sigma-Aldrich, St. Louis, MO, USA) at a dilution of 1:1000. Signal development was performed using HRP–conjugated secondary AB at a dilution of 1:10.000: anti-sheep HRP (Sigma-Aldrich) and anti-mouse HRP (Sigma-Aldrich). Protein bands were visualized by WesternSure^®^ PREMIUM Chemiluminescent substrate (LI-COR, Lincoln, NE, USA) using the C-DiGit^®^ Blot Scanner (LI-COR) and Image Studio^TM^ Lite Version 5.2 software (LI-COR).

### 2.4. Statistical Analysis

Microsoft Excel Version 16.16.27 (Microsoft, Redmond, WA, USA) and GraphPad Prism Version 9.1.0. (GraphPad Software, San Diego, CA, USA) were used for statistical analyses and constructing figures. 

## 3. Results

### 3.1. Immunohistochemistry

**Figure 1:**
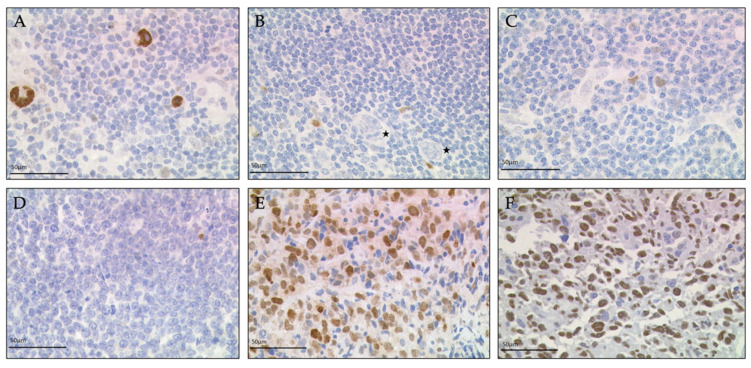
Characterization of BATF3-expression in tumor cells of classical Hodgkin lymphoma (cHL), nodular lymphocyte predominant Hodgkin lymphoma (NLPHL) and Non-Hodgkin lymphoma (NHL). Strong BATF3 expression in Hodgkin/Reed-Sternberg (HRS) cells of cHL (**A**). No BATF3 expression in lymphocyte predominant cells (asterisks) of NLPHL (**B**) and in tumor cells of follicular lymphoma (FL) (**C**) and Burkitt lymphoma (BL) (**D**). Tumor cells of primary mediastinal large B-cell lymphoma (PMBL) (**E**) showed moderate BATF3 expression, tumor cells of anaplastic large cell lymphoma (ALCL) show strong BATF3 expression (**F**). Calculated BATF3-scores were 300 (**A**,**F**), 180 (**E**) and 0 (**B**–**D**).

We observed BATF3-expressing tumor cells in 100 of the 218 (*n* = 100/218) lymphoma samples distributed across 11 of the 14 (*n* = 11/14) entities examined. Strong BATF3 expression was a constitutive feature in cHL (*n* = 53/53) and in ALCL (*n* = 3/3). Varying degrees of BATF3 expression were observed in primary mediastinal large B-cell lymphoma (PMBL) (*n* = 14/15), diffuse large B-cell lymphoma (DLBCL) (*n* = 7/22), T-cell/histiocyte-rich large B-cell lymphoma (THRLBL) (*n* = 3/3), follicular lymphoma (FL) (*n* = 5/22), chronic lymphocytic leukemia/small lymphocytic lymphoma (CLL) (*n* =1/15), plasma cell neoplasm (PCN) (*n* = 1/15), peripheral T-cell lymphoma (PTCL) (*n* = 6/12), intestinal T-cell lymphoma (ITCL) (*n* = 2/2) and NLPHL (*n* = 7/16) (Figure 1). 

**Figure 2:**
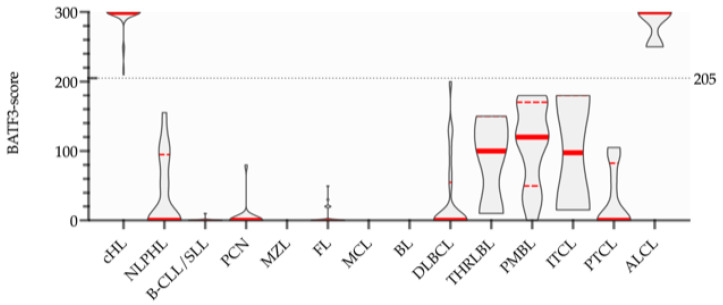
Truncated violin plots of BATF3-scores. Samples from CHL and ALCL showed constitutively high BATF3-scores, which could be clearly differentiated from those of the other lymphoma entities. An apparent threshold of 205 is indicated. Median (**—**) and Quartiles (**---**) are highlighted.

BATF3-scoring was used to evaluate the BATF3 expression semi-quantitatively and to highlight the differences in the BATF3 expression between the individual lymphoma entities. BATF3-scores were constitutively high in cHL and ALCL and constitutively low in CLL, FL, MCL, MZL, PCN and BL. An apparent threshold of 205 separated cHL and ALCL from the other lymphoma entities. DLBCL, PMBL, THRLBL, PTCL, ITCL and NLPHL showed low to moderate BATF3-scores (Figure 2). 

### 3.2. Western Blotting

**Figure 3:**
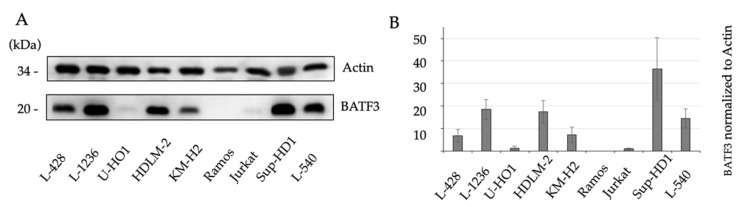
Characterization of BATF3 expression in cHL cell lines. Western blots of cell lysates from cHL (L-428, L-1236, U-HO1, HDLM-2, KM-H2, Sup-HD1, L-540), T-cell lymphoblastic lymphoma/Leukemia (T-LBL/ALL) (Jurkat) and BL (Ramos) were probed with anti-BATF3 AB and anti-β-actin AB. Anti-BATF3 AB labeled the 17-kDa BATF3-protein in cHL and in T-LBL/ALL, while BL cells were not reactive (**A**). Quantification of the amount of BATF3 protein as depicted in A. Mean value of 3 independent experiments (see Figure S1 in Supplementary Materials) is shown (**B**).

Western blot analysis confirmed BATF3 protein expression in cHL. Anti-BATF3 AB labeled the 17-kDa BATF3 protein in cell lysates from cHL (L-428, L-1236, U-HO1, HDLM-2, KM-H2, Sup-HD1, L-540) and T-LBL/ALL (Jurkat), while cell lysates from BL (Ramos) were not reactive (Figure 3). 

## 4. Discussion

This study addresses the usefulness of BATF3 as an ancillary IHC marker for the diagnosis of cHL. We performed immunohistochemistry and analyzed the BATF3 protein expression in lymphoma cells on 218 lymphoma samples belonging to 14 different lymphoma entities. BATF3 expression was a constitutive feature of cHL and observed in all samples investigated (*n* = 53/53). 

Our findings are consistent with gene expression studies that demonstrated constitutively high levels of BATF3 mRNA in cHL and ALCL [17,18,19,20,21,22,23,24]. In some of these studies, IHC was carried out and BATF3 expression was reported in all (Schleussner et al. (*n* = 8/8)) or in the majority (Lollies et al. (*n* = 21/30) and Vrzalikova et al. (*n* = 36/52)) of the analyzed cHL samples [20,21,22]. We used Western Blot analysis to confirm the BATF3 protein expression in cHL (*n* = 7/7). The cHL cell lines examined showed variable amounts of BATF3 protein (Figure 3). Different levels of BATF3 protein expression could explain the differences in the relative number of the BATF3-positive samples reported in the respective IHC studies [20,21,22], since the IHC protocols and evaluation methods used may have different detection sensitivities. Since we did not observe BATF3-negative cHL samples, we did not systematically query for EBV status, although one group (Lollies et al) reported lower BATF3 expression in EBV-negative HRS cells [20]. Thus, we show that BATF3 expression in cHL can be reliably demonstrated by immunohistochemistry.

BATF3-expressing tumor cells were observed in 100 of the 218 lymphoma samples and were not limited to cHL. In particular, tumor cells of those lymphoma entities, whose diagnostic differentiation from cHL is sometimes difficult (ALCL, DLBCL, PMBL, THRLBL, PTCL and NLPHL) showed increased BATF3 expression. Even so, by evaluating the BATF3 expression in analogy to the H-score method [29], we were able to demonstrate clear differences in the BATF3-scores between the individual lymphoma entities. However, the apparent threshold of 205 that separated cHL and ALCL from the other lymphoma entities was data driven and has not been validated so far. We are the first to report on the BATF3 expression status in ITCL, PCN and MZL, but some of the lymphoma entities in this study included only a few samples. Therefore, more extensive investigations with larger numbers of samples are necessary in order to substantiate the differences in the BATF3-scores. Thus, for diagnosing cHL, BATF3 immunohistochemistry should be used in conjunction with BATF3-scoring in order to obtain an acceptable diagnostic specificity.

## 5. Conclusions

We consider BATF3 to be a useful IHC marker for the diagnosis of cHL. BATF3 expression is a constitutive feature of cHL and can be reliably demonstrated by IHC staining. BATF3-positivity can confirm the diagnosis of cHL in cases where HRS cells display an atypical immunophenotype or the specimen's properties limit the use of other IHC markers. Moreover BATF3-scoring can help to differentiate cHL from DLBCL, PMBL, THRLBL, PTCL and NLPHL.

## Data Availability

The data presented in this study are available on request from the corresponding author.

## Supplementary Materials

The following are available online at https://www.mdpi.com/article/10.3390/diagnostics11061123/s1, Figure S1: Western blot analysis of BATF3 expression in cHL cell lines.
