# Supplementary material for: Usefulness of BATF3 Immunohistochemistry in Diagnosing Classical Hodgkin Lymphoma
Source: Diagnostics (Basel). 2021 Jun 20;11(6):1123. doi: 10.3390/diagnostics11061123 (PMC8234195; doi:10.3390/diagnostics11061123)
Supplement: Supplementary file 1 [file diagnostics-11-01123-s001.zip › diagnostics-1255210-supplementary.pdf]

Supplementary Materials: The following are available online at www.mdpi.com/xxx/s1, Figure S1: Western blot analysis of BATF3 expression in cHL cell lines.

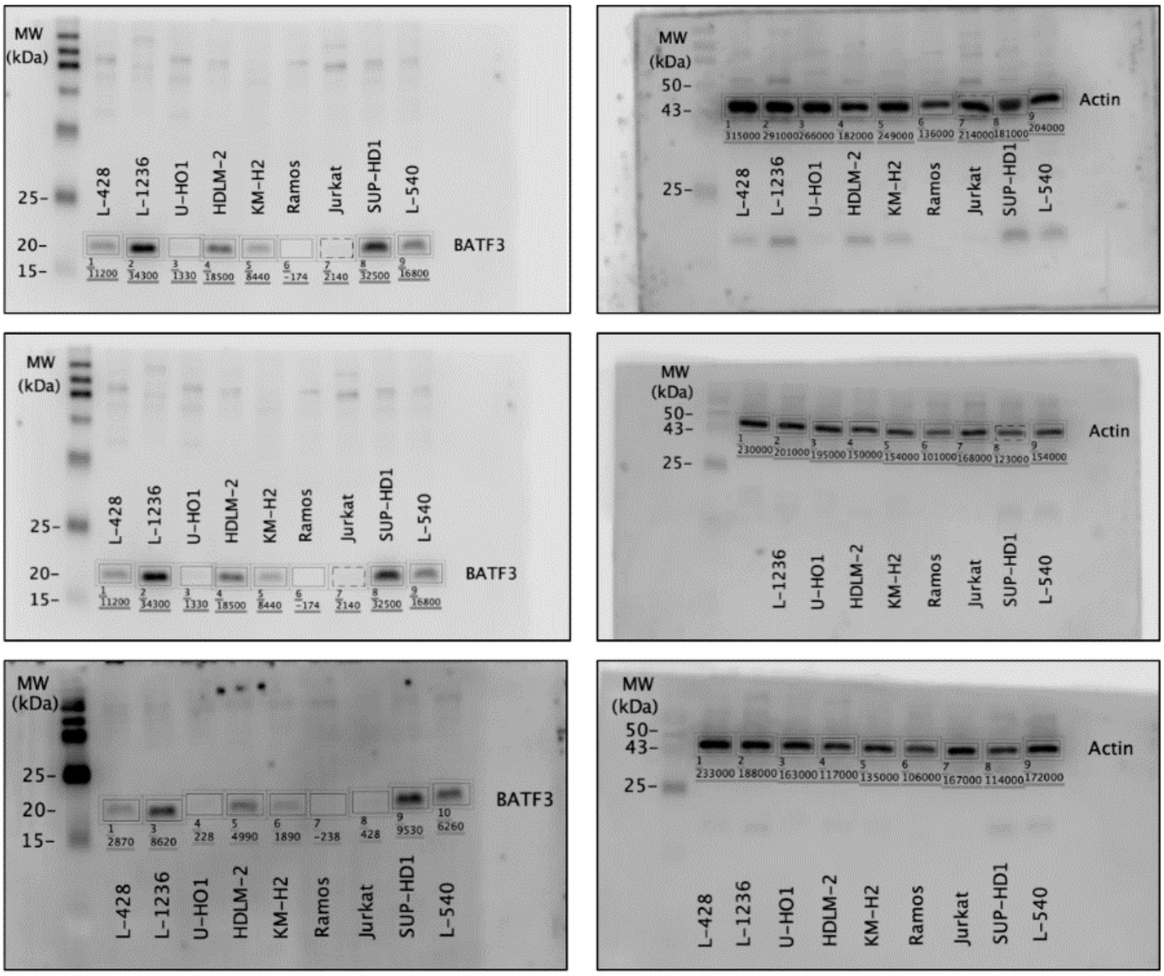

Figure S1. Western blot analysis of BATF3 expression in cHL cell lines. Western blots of cell lysates from cHL (L-428, L-1236, U-HO1, HDLM-2, KM-H2, Sup-HD1, L-540), T-LBL/ALL (Jurkat) and BL (Ramos) were probed with anti-BATF3 AB and anti-β-actin AB. 3 independent experiments are shown that include densitometry readings/intensity ratio of each band. Anti-BATF3 AB labeled the 17-kDa BATF3-protein in cHL and in T-LBL/ALL, while BL cells were not reactive.
